# The Impact of Emotional Intelligence on Domain-Specific Creativity: The Mediating Role of Resilience and the Moderating Effects of Gratitude

**DOI:** 10.3390/jintelligence10040115

**Published:** 2022-11-27

**Authors:** Dandan Tong, Hanxiao Kang, Minghui Li, Junyi Yang, Peng Lu, Xiaochun Xie

**Affiliations:** 1 School of Psychology, Northwest Normal University, Lanzhou 730070, China; 2 Key Laboratory of Behavioral and Mental Health of Gansu Province, Northwest Normal University, Lanzhou 730070, China; 3 School of Education Science, Xinyang Normal University, Xinyang 464000, China; 4 Institute of Mathematics and Computer Science, Northwest Minzu University, Lanzhou 730070, China; 5 School of Psychology, Northeast Normal University, Changchun 130024, China

**Keywords:** emotional intelligence, domain-general creativity, domain-specific creativity, resilience, gratitude

## Abstract

Creativity incorporates both domain-general and domain-specific ideas. While previous studies have explored the impact of emotional intelligence (EI) on creativity in both domains, a consensus has not been reached, and the mechanism is currently unclear. In the present study, we examined which aspect of creativity EI was most strongly associated with in a group of undergraduates. Moreover, we explored the moderated mediation effect between EI and domain-specific creativity. In Study 1, 532 undergraduates completed questionnaires measuring EI, convergent and divergent creative thinking, and creative achievement. The results revealed that the most reliable positive correlations were between EI and domain-specific creativity. In Study 2, 926 undergraduates completed measurements of EI, resilience, gratitude, and creative achievement. The results revealed that resilience mediates the relationship between EI and creative achievement. Furthermore, gratitude moderated the indirect effect of EI on creative achievement through resilience. The indirect effect of EI on creative achievement was stronger for high-gratitude individuals than for low-gratitude individuals. This orientation and other results are discussed. Overall, our findings add further nuance to the relationship between EI and creativity in different domains. This study serves as a basis for other contributions aligned with these concepts.

## 1. Introduction

Creativity is the ability of an individual to produce a novel and appropriate product. It is commonly seen as the cornerstone of human progress (Jung et al. 2013; Sternberg and Lubart 1999). Is creativity the same across different domains? The amusement park theoretical model of creativity (APT Model) states that creativity incorporates both domain-general and domain-specific creativity (Baer and Kaufman 2017). By differentiating initial requirements, general thematic areas, domains, and micro-domains, the authors clarified the debates that highly creative individuals tend to be creative in different or specific domains. For creative thinking across different domains, which reflects the ability to break mental sets and formulate new ideas, divergent thinking (DT) and convergent thinking (CT) are two notable components. Creative behavior and achievement in domain-specific creativity are defined as the sum of creative products and long-term creative behaviors in multiple/specific creative domains (such as art, science, etc.) generated by an individual (Carson et al. 2005). Moreover, different scales have been developed to measure domain-general and domain-specific creativity. For instance, the Torrance Tests of Creative Thinking battery (TTCT) (Torrance 1966) and the alternative uses task (AUT) were typically used to measure DT, and the remote associates test (RAT) (Mednick 1967) was used to measure CT. These tests incorporate objective scoring criteria and lead to uniform results. The Kaufman domains of creativity scale (K-DCS) and the creative achievement test (CAQ) are frequently employed to measure overall creative behavior and achievement in domain-specific creativity (Kaufman 2012). The CAQ, which contains multiple domains of creativity achievements, has become the most widely used tool because of its objective scoring criteria and high ecological efficiency (Carson et al. 2005; Karwowski et al. 2021; Chen et al. 2014). 

### 1.1. Emotional Intelligence and Creativity

The personal creativity function model shows that personality is closely related to and is an important factor affecting creativity (Feist 2019; Hornberg and Reiter-Palmon 2017). Exploring personality traits associated with creativity helps to reveal its essential characteristics (Tambunan et al. 2022). Further, regardless of the domain, individual trait factors can have an impact on creativity. Therefore, it is important to explore the differences between individual traits of domain-general creativity and domain-specific creativity, as well as their underlying mechanisms (Nori et al. 2018). An increasing number of researchers in the field have focused on the link between individual differences in personality traits (e.g., openness to experience and conscientiousness) and creativity at all ends of domain specificity and domain generality (Feist 1998; Puryear et al. 2017). Emotional intelligence (EI) refers to an individual’s ability to manage and use emotions to guide their thoughts and actions. The four-branch model further refines the concept of EI, which includes four aspects: understanding one’s own and others’ emotions, facilitating cognition through emotions, perceiving emotional information, and controlling one’s own and others’ emotions (Mayer et al. 2004). EI is an important predictor of adaptation and life success, including physical and mental health, social relationships, and job performance (Salovey and Mayer 1990; Hoffmann et al. 2020).

Salovey and Mayer (1990) originally asserted that EI helps the processes of intuition and stimulates creative thinking. Subsequently, numerous studies have found significant but complex results regarding the relationship between EI, domain specific, and domain-general creativity. For example, studies using the Verbal Test of Creative Thinking and AUT on adolescents have found that EI is not associated with domain-general creativity (Mishra 2014; Neubauer et al. 2018). Conversely, another study used the AUT in gifted and normal adults to demonstrate that EI might be associated with domain-general creativity (Ahangar Ghorbani et al. 2022). A training study using drama pedagogy training (DPT) among school-going children aged 9–11 years similarly found an association between EI and creativity, that is, through an increase in positive emotions, creativity was also promoted (Zenasni and Lubart 2011; Celume et al. 2019). Moreover, one study used the adapted K-DOCS to measure the domain-specific creativity of gifted teenagers and found that the total score of EI was only related to everyday creativity (Şahin et al. 2016). However, further research has emphasized that creativity in specialized fields is more strongly associated with EI. For example, researchers have used the emotional creativity inventory (ECI) (Averill 1999), the 3-item scale (Oldham and Cummings 1996), and K-DOCS (Tu et al. 2020) to test the relationship between EI and emotional creativity (EC), everyday creativity, academic performance, scientific/mechanical creativity, and artistic creativity among college students, corporate employees, and gifted youths. 

Although many studies have explored the relationship between EI and creativity, gaps remain (Tu et al. 2020; Xu et al. 2019). For example, in terms of tool testing, existing studies have primarily used K-DOCS to measure domain-specific creativity. The K-DOCS is a self-assessment tool based on individuals’ subjective feelings and can vary with a person’s capacity to accurately judge their abilities. This often results in the problem of common method variance and makes research conclusions less comparable (Ng and Feldman 2012; Kaufman 2019). In terms of research subjects, previous studies have focused on employees, gifted adults, and adolescents (Eyni et al. 2021; Zenasni and Lubart 2009; Şahin 2016), such an arbitrary selection of participants may ignore individual differences, making the related findings less convincing (Ling et al. 2016). Researchers have shown that creativity helps university students to increase their competitiveness in the labor market and make more novel and effective decisions in their future work (Denysenko 2018). However, not all universities are equipped with creative ability, which is why the study of creativity among university students is particularly important (Denysenko 2018; Ilyashenko et al. 2019). Finally, sample size moderates the link between EI and creativity. The sample sizes of existing studies were mostly between 30 and 50 participants, such small sample sizes may limit the statistical power of the research results (Xu et al. 2019; Angela and Caterina 2020). Based on the findings summarized above, Study 1 aimed to use DT and CT to measure domain-general creativity. The CAQ was used to evaluate domain-specific creativity in the same study and explore the relationship between EI and creativity in a large sample of college students.

Based on cognitive processing theory (Mayer et al. 2008), EI and creative thinking represent completely different cognitive abilities, which may be the reason for low correlation levels of EI and domain-general creativity (Zenasni and Lubart 2009; Sordia et al. 2019). However, based on Emotional Motivation Theory (Gable and Harmon-Jones 2010), people with high EI are more competent at sustaining and using positive affect. They are also more capable of channeling negative affect into change-oriented thinking processes, thus, probably generating creative solutions. Studies have explored the mechanisms underlying the influence of EI on creativity in detail. People with high EI possess the ability to identify, understand, manage, and utilize emotions, which helps them identify problems more appropriately, obtain problem-solving information in a positive and open manner, avoid negative emotions, and enhance their intrinsic motivation to think about facilitating problem solving, which ultimately promotes creative output (Zhou and George 2003; Lea et al. 2018). In addition, emotional regulation, a component of EI, has been associated with employment creativity by maintaining positive affect (Parke et al. 2015). Therefore, EI’s relationship with domain-specific creativity may benefit more from affect links than from the similarity of cognitive constructs. Furthermore, a meta-analysis showed that most domain-specific creativity measures are based on subjective reports. This measurement mode may have resulted in a stronger relationship between EI and domain-specific creativity (Xu et al. 2019). For example, Tu et al. (2020) identified a stronger EI and domain-specific creativity correlation when domain-specific creativity was measured using the Kaufman domains of the creativity scale. A weaker correlation was found when domain-generality creativity was measured using the abbreviated Torrance test for adults. In this study, the use of the CAQ to measure domain-specific creativity was a subjective measure compared with the DT and CT tests to measure domain-general creativity. Based on the literature review, we propose the following hypothesis:

**Hypothesis** **1.** *Emotional intelligence is significantly correlated with a domain-specific measure of creative achievement, but not with a domain-general measure of divergent and convergent thinking.*

Two issues remain unresolved in previous studies that have explored the relationship between EI and domain-specific creativity. First, the direct effect of EI on domain-specific creativity cannot help readers to understand how EI exacerbates domain-specific creativity. Although these studies explored the effects of EI on sense of humor, generosity, openness to experience, and confidence, they failed to link this to affect resilience (Carmeli et al. 2014; Ahangar Ghorbani et al. 2022; Winton and Sabol 2022). Thus, in this study, we examined the mechanism resilience mediates the relationship between EI and domain-specific creativity. An integrated mediation model could provide a better understanding of how EI relates to domain-specific creativity by maintaining a positive state of stability. Second, previous meta-analysis and heterogeneous correlation coefficients between EI and domain-specific creativity suggest that some potential moderators might influence these relationships (Xu et al. 2019). It is necessary to explore a moderator that strengthens the effects of protective factors such as gratitude. Therefore, in Study 2, a moderated mediation model could simultaneously reflect how and when EI may affect domain-specific creativity.

### 1.2. Emotional Intelligence, Resilience, and Domain-Specific Creativity

Resilience refers to an individual’s ability to recover to a positive state after experiencing significant stress or adversity (Masten et al. 1990). It can be viewed as an extremely useful personal resource that helps individuals cope with continually changing lives (Feldman 2020). According to Emotional Motivation Theory (Gable and Harmon-Jones 2010), individuals who experience positive emotions tend to maintain a positive state of stability and improve creative problem solving. The stabilization of resilience may play an important role in this process (Avey et al. 2010; Cai et al. 2019). Research has shown that individuals with high EI can better regulate their emotions and promote their development in a positive direction, which is conducive to maintaining a higher level of resilience (Alegre and Benson 2010; MacCann et al. 2020). For instance, one study found that high emotion regulation (a component of EI) increased resilience (Polizzi and Lynn 2021). Moreover, previous studies have found that highly resilient individuals have more psychological resources and are open to new experiences, leading to a greater positive adaptive state and cognitive flexibility, promoting creative thought and behaviors (Conley et al. 2016; Chin et al. 2018; Arici-Ozcan et al. 2019). For example, employees with high resilience have greater psychological resources, which can enhance specific creativity (Yu et al. 2019). Thus, a high level of EI results in a more pleasant experience, which is beneficial to the development of psychological elasticity. Through psychological resources, resilience improves domain-specific creativity (Li et al. 2022). Therefore, resilience affected by EI can, to a certain extent, affect domain-specific creativity. Based on this, we developed the following hypothesis:

**Hypothesis** **2.** *Resilience mediates the effect of emotional intelligence on domain-specific creativity.*

### 1.3. Emotional Intelligence, Resilience, Gratitude, and Domain-Specific Creativity

Gratitude refers to an individual’s psychological tendency to return to others with an appreciative attitude when experiencing positive results (McCullough et al. 2002). Gratitude is a significant resource for individuals, as it helps to construct positive relationships and nurture appreciation for life’s positive aspects (Wood et al. 2010; Di Fabio et al. 2017). Previous studies have shown that gratitude is closely related to EI and domain-specific creativity (Arnout and Almoied 2021; Szcześniak et al. 2020). The broad-build theory of positive emotions posits that gratitude helps broaden thought patterns, build positive and lasting social and psychological resources, and eliminate the effects of negative emotions (Fredrickson 2004; Tugade et al. 2004). A substantial body of research has established gratitude as a protective factor of creativity (Wu et al. 2020; Pillay and Park 2017). The protective-enhancing hypothesis maintains that one protective factor enhances the advantages of another (Fergus and Zimmerman 2005; Li et al. 2013). Although not directly related to creativity, one study demonstrated a similar protective effect. They examined the moderating relationship of gratitude in emotional regulation and burnout (Guan and Jepsen 2020). This study showed that a high level of gratitude could modulate the impact of emotion on burnout by building positive psychological and social resources. Research has shown that individuals with a high level of gratitude perceive more support and are more psychologically secure, thereby improving their resilience (Fredrickson and Joiner 2002; Kim et al. 2018; Ozbay et al. 2008). In addition, the broad-build theory of positive emotions (Fredrickson 2001) also emphasizes that positive emotions can promote the development of creativity (Wu et al. 2020; Li et al. 2017) by expanding cognitive maps and enhancing thought patterns (Wood et al. 2010; Folkman and Moskowitz 2000; Tugade et al. 2004). For example, a previous study confirmed that highly grateful individuals are emotionally stable, actively develop information processing, and contribute to team creativity (Huang and Lu 2020). Therefore, individuals with a high level of gratitude convert EI to creativity through a combination of emotional and cognitive processes. A series of emotional and cognitive changes generated by gratitude will impact the process by which EI mediates creativity in specific fields through resilience. Based on this, we propose the following hypothesis:

**Hypothesis** **3.** *Gratitude moderates the effects of emotional intelligence on domain-specific creativity through resilience.*

### 1.4. The Present Study

We evaluated these hypotheses in Study 1. Questionnaires measuring EI, DT, CT, and CAQ were used to explore the relationship between EI and domain-general and domain-specific creativity. Measures of EI, resilience, gratitude, and CAQ were constructed to evaluate the moderated mediation model in Study 2.

## 2. Study 1

### 2.1. Materials and Methods

#### 2.1.1. Participants

A total of 548 individuals from two universities in northwest China participated in the study. The data of sixteen participants were removed from the data set because of outlying scores or incompletion of the questionnaires. Of the remaining 532 participants, 310 were females, mean age = 19.49 years, SD = 0.99; 222 were males, mean age = 19.25 years, SD = 1.08. Convenience sampling was the selected sampling method. The participants were given a small gift after completing all tasks. Moreover, we used verbal and figure TTCT to further explore the relationship between EI and creative thinking and eliminate the possible impact of a single AUT on the results. Datasets from 273 participants (124 males, mean age = 20.12 years, SD = 1.32) were acquired from the Gene-Brain-Behavior (GBB) Project, an ongoing longitudinal project from the Creativity and Affective Neuroscience Lab (CANL) at Southwest University. 

#### 2.1.2. Materials

##### Emotional Intelligence Scale

The Schutte Self-Report Emotional Intelligence Scale (SSREIS), developed by Schutte et al. and adapted into a Chinese version by Huang et al. (Schutte et al. 1998; Huang et al. 2008), was used to assess the participants’ EI. The scale includes four subscales: Monitor of Emotions (e.g., “I know why my emotions change”), Utilization of Emotions (e.g., “When I am in a positive mood, I can come up with new ideas”), Social Ability (e.g., “I help other people feel better when they are down”), Appraisal of Emotions (e.g., “I am aware of the non-verbal messages other people send”). Participants rated the items using a 5-point scale from 1 = not true of me to 7 = very often true of me. In the present study, Cronbach’s α for the scale was 0.74.

##### Creative Achievement Questionnaire

This study used a self-reporting Creative Achievement Questionnaire to measure the participants’ creative achievement (Carson et al. 2005). This questionnaire includes three parts and contains 96 items. The participants were required to indicate that they self-perceived more ability or talent than others in the listed 13 areas with a checkmark in part one. Part two lists concrete achievements in the 10 domains (e.g., “visual arts, music, dance, creative writing, architectural design, humor, theater and film, culinary arts, inventions, and scientific inquiry”). Each domain includes a “no achievement” item (e.g., “I have no training or talent in this field”), a “training” item (e.g., “I have been trained in this field”) and six “ascending achievement” items (e.g., “I have received a number of awards in the field”). The participants ranked domains such as “Inventions” from 0 = I have no training or recognized talent in this area to 7 = One of my inventions was sold to a manufacturing company. In items marked with an asterisk, the participants were asked to indicate how many times they reached each achievement. Part three allows participants to add other creative achievements in addition to the 10 areas mentioned above. The total score of the 10 different domains from part two indicates participants’ creative achievement. In the present study, Cronbach’s α for the scale was 0.80.

##### Unusual Uses Task

The Unusual Uses Task from The Torrance Tests of Creative Thinking (TTCT) developed by (Torrance 1966) was used to measure the participants’ divergent thinking. The participants were asked to generate as many interesting, uniqueness, and uncommon uses as possible for cardboard boxes without limitation of the size and number in 10 min (e.g., “unusual uses for cardboard boxes”). Responses for the tasks were scored via originality (the degree of uniqueness of each item), fluency (the total number of meaningful ideas), and flexibility (the number of categories of relevant responses). Based on the criteria table, the responses were independently rated by two trained raters. The total score was summed in terms of originality, fluency, flexibility, and overallDT. The interrater scoring reliability was 0.93 for originality, 0.97 for fluency, 0.92 for flexibility, and 0.95 for overall DT.

##### Remote Association Test

The Remote Association Test developed by (Mednick and Mednick 1962) was utilized to assess convergent thinking. Each item consists of three Chinese “clue” words that can be associated with a “solution” word to form a compound word or specific a semantic association. All 25 items are constructed in such a way that only a solution is possible. For example, the solution to the three words “pai(拍), mai(买), fan(贩)” is “mai” (卖) by forming the compound word (拍卖, 买卖, 贩卖), and the problem “mo(没), zuo(坐), cun(村)” is solved by “luo” (落) by forming the compound word (没落, 坐落, 村落). The total score of the 25 items indicates participants’ ability to produce a more remotely related word. In the present study, Cronbach’s α for the scale was 0.80.

##### Torrance Test of Creative Thinking 

The Torrance Test of Creative Thinking (TTCT) contains figural and verbal tasks. It is commonly used to assess divergent thinking and has an extensive application in the test of creativity (Torrance 1966; Kim 2006). The verbal TTCT involves seven types of activities: to generate questions, causes and consequences in response to a scenario (tasks 1–3); to improve a toy elephant (task 4); to generate uses for cardboard boxes (task 5); to think of questions relating to a carton (task 6); to imagine the consequences of an imaginary scenario (task 7). The figural TTCT comprises three tasks. The participants were asked to construct a picture or as many as possible objects based on an ellipse (task 1), 10 incomplete figures (task 2), and vertical lines (task 3). In the present study, Cronbach’s α for the verbal TTCT scale was 0.89, and the figural TTCT scale was 0.96.

### 2.2. Results

#### 2.2.1. The Correlations among EI, UUT, RAT, and CAQ

The descriptive statistics and correlations between EI and four subscales are presented in Table 1. Since the subscales of emotional intelligence are highly correlated with the total score, only the total score was included in the analysis. Descriptive statistics and correlations between EI, UUT, and RAT are presented in Table 2. We also examined the relationship between EI and CAQ in ten domains—see Table 3. We found almost all correlations to be significant, except for those sub-dimensions of creative achievement (dance, architectural and theater). These results show that EI is more related to domain-specific creativity.

#### 2.2.2. The Correlations among EI, TTCT, and RAT

The descriptive statistics and correlations between the EI, TTCT, RAT, and CAQ are presented in Table 4. These results demonstrate that EI is congruous positively correlated with creative achievement but almost not with verbal and figural divergent thinking, except the flexible verbal score.

## 3. Study 2

### 3.1. Materials and Methods

#### 3.1.1. Participants

A total of 955 individuals from three universities in northeast and northwest China participated in the study. The data of twenty-nine participants were removed from the data set because of outlying scores or incompletion of the questionnaires. Among the remaining 926 participants were 301 males with an average age of 19.41 ± 1.14 years and 625 females with an average age of 19.55 ± 0.99 years. Convenience sampling was the selected sampling method. The participants completed the questionnaires in quiet classrooms following instructions given by the researchers, and they were given a small gift after completing all tasks.

#### 3.1.2. Materials

##### Connor-Davidson Resilience Scale

The 10 item Connor-Davidson Resilience Scale (CD-RISC-10) developed by (Campbell-Sills and Stein 2007) was used to assess participants’ resilience. This 10-item (e.g., “I can adapt to changes.”) abbreviated scale is widely used and has good internal consistency and stable test-retest reliability in Chinese samples (Wang et al. 2010; Cheng et al. 2020). Items were rated on a 5-point scale from 0 = not at all to 4 = a lot. A higher average score indicates that an individual can more successfully deal with adversity. In the present study, Cronbach’s α for the scale was 0.87. 

##### The Gratitude Questionnaire

The Gratitude Questionnaire, developed by McCullough et al. and adapted into a Chinese version by Chen, was used to assess participants’ dispositional gratitude (Chen et al. 2009; McCullough et al. 2002). This scale consists of six items (e.g., “There are so many things in my life that I am grateful for.”). Participants rated items on a 7-point Likert scale from 1 = completely not true to 7 = completely true. A higher score represents higher dispositional gratitude in a participant. In the present study, Cronbach’s α for the scale was 0.70.

The Emotional Intelligence Scale and Creative Achievement questionnaires were used to assess the participants’ EI and creative achievement, respectively. In the present study, Cronbach’s α for the Emotional Intelligence Scale was 0.70 and the Creative Achievement Questionnaire was 0.79.

#### 3.1.3. Statistical Analysis Methods

The distribution of indirect effects is often not normal. The Sobel test provides biased results because it is based on a normal distribution (Hayes 2018). Therefore, this study used Model 4 in the PROCESS template, SPSS Marco (Version 3.4, Hayes 2018), to evaluate the mediation effect, and Model 59 to evaluate the moderated mediation effect. All continuous variables were standardized before mediation analyses and randomly sampled 5000 times from the original data using the bootstrapping method. In addition, gender and age were controlled for in the regressions. 

### 3.2. Results

#### 3.2.1. Descriptive Statistics and Correlations

The descriptive statistics and correlations are shown in Table 5; all correlations were found to be significant.

#### 3.2.2. Mediation Analyses

**Figure 1:**
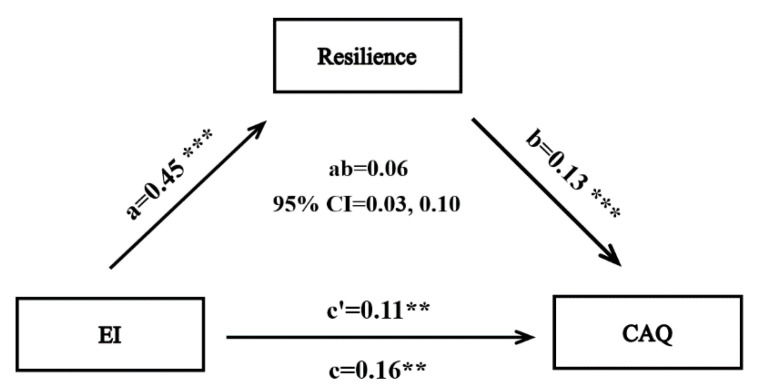
Mediation analyses. Note: Full items are listed by abbreviations. EI—emotional intelligence; CAQ—CAQ score. ** *p* < 0.01; *** *p* < 0.001.

The SPSS Macro PROCESS was used to test the mediating role of resilience in the relationship between EI and creative achievement. As shown in Figure 1, EI positively correlated with creative achievement (b = 0.16, *p* < 0.001) and resilience (b = 0.45, *p* < 0.001). With the addition of resilience in the regression model for creative achievement, the regression coefficient of EI was reduced to 0.11 (*p* < 0.01). Moreover, resilience positively correlated with creative achievement (b = 0.13, *p* < 0.001). Thus, this study found that resilience mediated the relationship between EI and creative achievement; specifically, the indirect effect was 0.06, 95% CI = [0.03, 0.10]; the direct effect was 0.11, 95% CI = [0.03, 0.18]. The indirect effect explained 35.35% variance of the total effect.

#### 3.2.3. Moderated Mediating Effect Test

**Figure 2:**
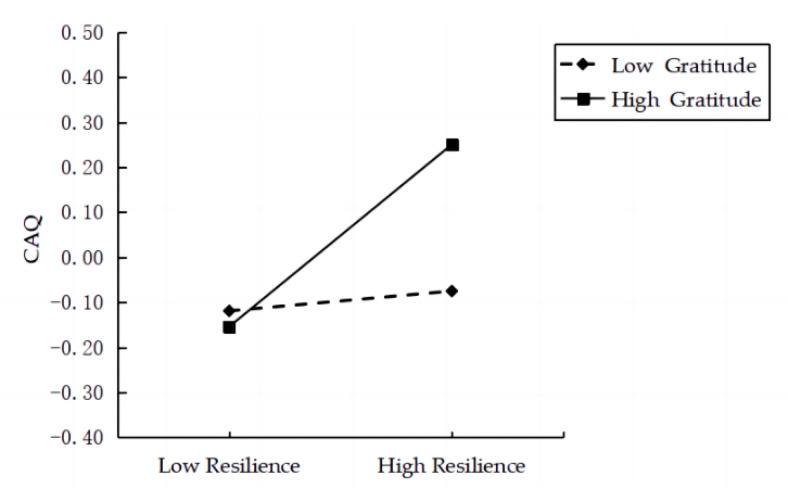
The moderating effect of gratitude on the relation between resilience and CAQ.

The moderating role of gratitude was tested using the PROCESS template. The results suggested that the interaction of resilience and gratitude positively predicted creative achievement (b = 0.09, *p* < 0.05); however, the interaction of EI and gratitude did not correlate with resilience (b = 0.04, *p* = 0.10). Table 6 shows the regression models. A simple slope test demonstrated that for low-gratitude individuals, the relationship between resilience and creative achievement was nonsignificant (bsimple = 0.02, *p* = 0.68). However, for high-gratitude individuals, the relationship between resilience and creative achievement was significant (bsimple = 0.20, *p* < 0.001). Figure 2 shows the interaction. Therefore, the mediating effect was only present for high-gratitude individuals, ab = 0.09, 95% CI = [0.03, 0.16]. These analyses indicate that gratitude moderated the indirect effect of EI on creative achievement for high-gratitude individuals, but not for low-gratitude individuals.

## 4. Discussion

In this study, we investigated the relationship between EI and domain-general and domain-specific creativity, as well as the mediating role of resilience and the moderating role of gratitude in the association between EI and domain-specific creativity. The results revealed the most reliable positive correlation between EI and creative achievement. More importantly, resilience played a mediating role in these associations and gratitude moderated the indirect effect. The indirect effect of EI on creative achievement was stronger for high-gratitude individuals than for low-gratitude ones.

### 4.1. The Association between Emotional Intelligence and Domain-General and Domain-Specific Creativity 

This study first explored the connection between EI and domain-general and domain-specific creativity among Chinese undergraduate students. This result is in line with Hypothesis 1 and the findings of a recent study (Hoffmann et al. 2020), which found that EI was more closely related to domain-specific creativity than domain-general creativity. Similarly, previous studies also found that while EI was rarely correlated with some aspects of DT or objective creativity tests, it was positively associated with domain-specific creativity and real-life creative problem-solving in a variety of samples (Tu et al. 2020; Mishra 2014; Yang et al. 2021). The subjective reports measurement model shows good reliability and validity and may result in a stronger relationship between EI and domain-specific creativity (Xu et al. 2019). One possible explanation for this discrepancy may be that EI is emotionally fundamental to achievement in a creative process (Parke et al. 2015; Petrides et al. 2007; Ivcevic and Hoffmann 2017). According to Emotional Motivation Theory, individuals with higher EI are more likely to perceive, understand, use, and regulate emotions (Trigueros et al. 2019). Furthermore, sustaining and using positive affect can improve creativity achievement by expanding the flexibility of thinking and responding to problems positively (Salovey and Mayer 1990; De Dreu et al. 2008; Lea et al. 2018). For example, a study of a group of college students proposed that the maintenance of positive emotions led to an increase in individual domain-specific creativity (measured using the K-DOCS) (Sharma et al. 2022). 

In addition, creative behavior or achievements in different domains such as art, science, and employment require different resources from diverse personal and environmental dimensions involving social facilitation mechanisms, such as social relationships and social effects, rather than creative thinking. For example, sociability is the sub-domain of EI, which refers to the traits of having the ability to effectively cope with problems and affect the emotions of other people (Quattropani et al. 2022). Sánchez-Ruiz et al. (2011) determined significant and slight correlations between TTCT (figural form) and sociability. In addition to this, Şahin et al. (2016) found that sociability is a significant predictor of scholarly, mechanical/scientific, artistic performance, self/every day and art domains’ creativity. Individuals with higher sociability are strong and effective in social relationships—for example, they are active and gregarious—which is closely related to the creative domain. For employment creativity, Castro et al. (2012) explored the interpersonal role of team leaders’ EI in fostering followers’ employment creativity by influencing interpersonal social relations. Moreover, in a group of nurses, EI is considered a personal resource that promotes active participation in work and support from different groups around them, thus, creating a direct impact on creativity in the work domain (Toyama and Mauno 2017). Therefore, individuals with high EI can ensure an emotionally well-documented affective state, flexible thinking, and concentration. They are also better at cultivating higher quality social interpersonal relationships and combining all the available resources. Thus, EI is more relevant to domain-specific creativity than to domain-general creativity.

Moreover, some studies are inconsistent with the findings of domain-general creativity from the present study. For example, using the AUT task, the previous study found that EI equally enhanced domain-general creativity. When coping with unsolved problems, individuals with higher EI are more flexible in their thinking and tend to consider problems from different perspectives, thus, finding unusual, original, and creative solutions (Akbari Chermahini and Hommel 2012; de Rooij et al. 2017). There are two possible explanations for this contradiction. The first is that people with a higher EI develop strong domain-specific creativity not only through cognitive flexibility, but also via affect and social facilitation mechanisms. Therefore, the results are always more robust than domain-general creativity. The second explanation may be more concerned with the various moderators. A previous meta-analysis study revealed that the link between EI and creativity was modulated by gender, employment status, and culture (Xu et al. 2019). For example, employment status moderated the link between EI and creativity; this association is stronger in employees than in students. Therefore, if these various moderator variables were not considered in the study, it is possible that the findings are contradictory. In addition, the results are relatively consistent with the findings of domain-specific creativity. The results from studies with different groups have shown a positive correlation between EI and domain-specific creativity among teenagers, college students, adults, and corporate employees (Ivcevic et al. 2021; Sharma et al. 2022; Tambunan et al. 2022; Mishra 2014). For example, using the K-DOCS and the five items in the creativity questionnaires to measure creativity in college students and adults, respectively, EI was shown to be positively associated with creativity, which is consistent with the results we obtained in the college student population (Xu et al. 2019).

### 4.2. The Mediating Role of Resilience

Our study used a mediation model to find that EI is positively related to domain-specific creativity through resilience. Consistent with previous researches and Hypothesis 2, EI was positively correlated with resilience (Magnano et al. 2016; Thomas and Zolkoski 2020). EI refers to the ability to perceive, appraise, express, regulate, and repair emotions accurately and effectively (Schneider et al. 2013; Armstrong et al. 2011). Individuals with high EI have adaptive emotion regulation strategies, better social relationships, and better adjustment outcomes when confronted with stressful situations (Fletcher and Sarkar 2012; Sarrionandia et al. 2018). Therefore, EI can contribute to an individual’s ability to better manage stress and develop resilience. In line with previous findings and Hypothesis 2, resilience is positively correlated with domain-specific creativity (Li et al. 2022; Hou et al. 2018; Wang et al. 2022). Resilience can be viewed as a set of justified psychological beliefs that can enhance the personal capacity for effective behavior and outcomes (Abbas and Raja 2015; Cenciotti et al. 2017). Individuals with high resilience generate higher levels of positive emotional stability, greater cognitive flexibility, and new ways of thinking that stimulate domain-specific creativity (Newman et al. 2018; Alessandri et al. 2018). In addition, creative behavior and achievements are often involved in social adversity. Individuals with higher levels of resilience are perceived as more confident and humorous and are likely to employ their psychological resources effectively to overcome the setbacks they confront (Yu et al. 2019). For example, because of a lack of psychological resources to deal with negative feedback on novel ideas, lower-resilience individuals may be less likely to ensure that they can convince their colleagues to recognize their value (Conley et al. 2016). Resilience often helps individuals to engage or react more positively to adversity, which motivates their endurance in the face of obstacles and leads to more persistence in creative exploration and behaviors (Li et al. 2022; Hou et al. 2018; Luthans et al. 2007). This facilitates creative achievements. According to Emotional Motivation Theory (Gable and Harmon-Jones 2010), indirect effects indicate that EI positively relates to creative achievement by improving individuals’ resilience. This model identifies resilience as a potential mechanism in the relationship between EI and domain-specific creativity. 

### 4.3. The Moderating Role of Gratitude

The moderating effect was in line with the protective-enhancing hypothesis: participants’ gratitude enhances the positive correlation between EI, resilience, and domain-specific creativity through the link between resilience and domain-specific creativity. Specifically, this correlation was only significant among individuals with higher gratitude levels. Previous studies have found that gratitude plays a protective role in creativity (Pillay et al. 2020). The results showed that individuals who feel grateful are more inclined to share existing information more thoughtfully and thoroughly and discuss ideas more actively, thus, increasing team creativity (Pillay and Park 2017). Another study showed that gratitude could significantly predict counselor creativity during counseling (Arnout and Almoied 2021). Based on the broad-build theory of positive emotions, a possible explanation for this protective aspect is that high gratitude can broaden individuals’ thought patterns and fortify their enduring psychological resources (Fredrickson 2004). This is because grateful individuals feel more thankful for positive results in others (McCullough et al. 2002; Fredrickson 2004). Resilience fosters personal creative achievement by leading to higher levels of positive emotional stability, greater cognitive flexibility, and more positive actions or reactions to adversity (Li et al. 2022; Newman et al. 2018). As a psychological resource, gratitude affects resilience (Kim et al. 2018; Unanue et al. 2019). For example, individuals with greater psychological resources can retain the effects of resilience (Hou et al. 2018; Newman et al. 2018). Highly grateful individuals broaden thought patterns, build positive and lasting psychological resources more easily, and accumulate supportive resources and communal relationships (Hou et al. 2018; Huang and Lu 2020), which can influence the effect of resilience on fostering personal creative achievement. Therefore, high gratitude facilitates the transformation of resilience into a creative achievement. However, it is difficult for individuals with low gratitude to broaden and build resources under the same conditions; hence, they cannot convert resilience into creative achievement.

### 4.4. Limitations and Future Directions

Future research should address these limitations. First, the present study aimed to evaluate trait EI effects rather than ability EI effects on domain general and domain-specific creativity. Therefore, the impact of EI ability on domain creativity has not yet been examined. Thus, subsequent studies could be repeated for ability-EI situations. Second, convenience sampling was used in this study. This sampling method may limit the generalizability of the findings and reduce the validity of the study. In future, random sampling should be used to better represent the population. Finally, this study used a self-report, cross-sectional design, making it difficult to deduce a causal relationship. Therefore, inferences regarding the analyzed relationships should be made with caution when explaining the results of this study. Future research should include longitudinal studies. Despite these limitations, this study still offers notable contributions to research exploring the moderated mediation effect of gratitude on the impact of EI on domain-specific creativity through resilience. Theoretically, this study draws a systematic picture of the mechanisms through which EI helps individuals maintain resilience for domain creativity. We build on the existing literature, which focuses on the affective, cognitive, and social mechanisms between EI and creativity (Zenasni and Lubart 2009; Castro et al. 2012). At a practical level, the present study suggests that EI may allow us to identify individuals who are more capable of gaining domain-specific creative achievement. EI may be an important variable to provide information on the selection and cultivation of creative students in various domains.

## Figures and Tables

**Table 1:** Descriptive statistics and correlations between EI and subscales.

Variables	M	SD	1	2	3	4	5
1. ME	3.56	0.64	1				
2. UE	3.98	0.45	0.26 **	1			
3. SA	3.99	0.46	0.29 **	0.41 **	1		
4. AE	3.64	0.63	0.31 **	0.31 **	0.34 **	1	
5. EI	3.80	0.38	0.73 **	0.67 **	0.69 **	0.69 **	1

Note: Full items are listed by abbreviations. ME—monitor of emotions; UE—utilization of emotions; SA—social ability; AE—appraisal of emotions; EI—emotional intelligence. ** *p* < 0.01.

**Table 2:** Descriptive statistics and correlations between EI and creativity thinking.

Variables	M	SD	1	2	3	4	5	6
1. EI	3.8	0.38	1					
2. Original	9.05	4.53	0.08	1				
3. Flexible	7.01	2.61	0.07	0.90 **	1			
4. Fluency	10.62	4.57	0.05	0.89 **	0.87 **	1		
5. UUT	8.89	3.75	0.07	0.97 **	0.95 **	0.96 **	1	
6. RAT	15.05	4.76	−0.04	0.04	0.05	0.08	0.06	1

Note: Full items are listed by abbreviations. EI—emotional intelligence; UUT—UUT score; RAT—RAT score. ** *p* < 0.01.

**Table 3:** Descriptive statistics and correlations between EI and creativity achievement.

Variables	M	SD	1	2	3	4	5	6	7	8	9	10	11
1. EI	3.80	0.38	1										
2. Visual	0.99	3.80	0.10 *	1									
3. music	0.39	2.06	0.10 *	0.57 **	1								
4. dance	0.29	1.59	0.05	0.17 **	0.69 **	1							
5. architectural	0.14	1.05	0.04	0.38 **	0.82 **	0.79 **	1						
6. writing	1.12	3.02	0.10 *	0.36 **	0.55 **	0.47 **	0.52 **	1					
7. humor	0.93	1.71	0.13 **	0.05	0.09 *	0.11 *	0.11 **	0.20 **	1				
8. invention	0.57	2.10	0.13 **	0.66 **	0.76 **	0.56 **	0.74 **	0.49 **	0.23 **	1			
9. scientific	0.24	0.78	0.12 **	0.42 **	0.23 **	0.06	0.14 **	0.37 **	0.45 **	0.45 **	1		
10. theater	0.14	0.92	0.07	0.13 **	0.53 **	0.46 **	0.49 **	0.41 **	0.11 *	0.38 **	0.11 *	1	
11. culinary	0.44	0.55	0.09 *	0.06	0.05	0.04	0.05	0.04	0.14 **	0.13 **	0.13 **	0.07	1
12. CAQ	0.53	1.20	0.14 **	0.72 **	0.86 **	0.65 **	0.78 **	0.74 **	0.33 **	0.88 **	0.51 **	0.51 **	0.15 **

Note: Full items are listed by abbreviations. EI—emotional intelligence; Visual—visual arts; architectural—architectural design; scientific—scientific inquiry; theater—theater and film; culinary—culinary arts; CAQ—CAQ score. * *p* < 0.05; ** *p* < 0.01.

**Table 4:** Descriptive statistics and correlations between EI and creativity.

Variables	M	SD	1	2	3	4	5	6	7	8	9	10	11
1. EI	3.84	0.35	1										
2. Original-V	45.96	17.95	0.11	1									
3. Flexible-V	26.71	7.77	0.13 *	0.83 **	1								
4. Fluency-V	57.64	20.93	0.07	0.91 **	0.83 **	1							
5. TTCT-V	43.43	14.92	0.10	0.97 **	0.90 **	0.98 **	1						
6. Original-F	7.83	3.91	0.06	0.24 **	0.18 **	0.22 **	0.23 **	1					
7. Flexible-F	6.23	2.71	0.06	0.19 **	0.17 **	0.18 **	0.19 **	0.84 **	1				
8. Fluency-F	6.24	2.70	0.05	0.23 **	0.15 *	0.20 **	0.21 **	0.89 **	0.88 **	1			
9. TTCT-F	6.77	2.96	0.06	0.23 **	0.18 **	0.21 **	0.22 **	0.96 **	0.94 **	0.96 **	1		
10. RAT	15.78	3.90	0.06	−0.02	−0.02	−0.06	−0.04	−0.02	−0.05	−0.03	−0.03	1	
11. CAQ	0.52	0.50	0.16 **	0.24 **	0.23 **	0.23 **	0.24 **	0.13 *	0.14 *	0.09	0.13 *	0.05	1

Note: Full items are listed by abbreviations. EI—emotional intelligence; V—verbal; F—figure; TTCT—TTCT score; RAT—RAT score; CAQ—CAQ score. * *p* < 0.05; ** *p* < 0.01.

**Table 5:** Descriptive statistics and correlations between variables.

Variables	M	SD	1	2	3	4
1. EI	3.78	0.36	1			
2. Resilience	3.45	0.55	0.46 **	1		
3. CAQ	0.54	1.05	0.16 **	0.18 **	1	
4. Gratitude	5.78	0.92	0.31 **	0.32 **	0.12 **	1

Note: Full items are listed by abbreviations. EI—emotional intelligence; CAQ—CAQ score. ** *p* < 0.01.

**Table 6:** Moderated mediating analyses.

	Model 1 (Criterion = CAQ)		Model 2 (Criterion = Resilience)	
	*B*	t	*B*	t
Gender	−0.03	−0.36	−0.33	−5.38
Age	−0.03	−0.90	0.01	0.44
EI	0.09	2.48 *	0.39	13.00 ***
Resilience	0.11	2.99 **		
Gratitude	0.07	2.02 *	0.21	6.89 ***
Interaction	0.09	2.43 *	0.04	1.64
R2	0.05		0.27	
F Conditional indirect effects at moderator values = M ± SD	7.02 b	Boot SE	66.84 Boot LLCI	Boot ULCI
M − SD (−1) M (0) M + SD (1)	0.01 0.04 0.09	0.01 0.02 0.03	−0.02 0.02 0.03	0.04 0.08 0.16

Note: Full items are listed by abbreviations. SES—socioeconomic status; EI—emotional intelligence; CAQ—CAQ score. * *p* < 0.05; ** *p* < 0.01; *** *p* < 0.001.

## Data Availability

The data generated and analyzed during the current study are available from the corresponding author on reasonable request.
